# A literature review of clinical tests for lumbar instability in low back pain: validity and applicability in clinical practice

**DOI:** 10.1186/s12998-015-0058-7

**Published:** 2015-04-08

**Authors:** Silvano Ferrari, Tiziana Manni, Francesca Bonetti, Jorge Hugo Villafañe, Carla Vanti

**Affiliations:** Master of Manual Therapy and Musculoskeletal Rehabilitation, Department of Molecular Medicine, University of Padova, Padova, Italy; Physical Therapy Private Practice, Treviso, Italy; Physioup Physical Therapy Private Practice, Rome, Italy; IRCCS Don Gnocchi Foundation, Milan, Italy

**Keywords:** Joint instability, Lumbar instability, Low back pain, Physical examination, Reproducibility of results, Prone instability test, Passive lumbar extension test, Aberrant movements pattern, Posterior shear test

## Abstract

**Background:**

Several clinical tests have been proposed on low back pain (LBP), but their usefulness in detecting lumbar instability is not yet clear. The objective of this literature review was to investigate the clinical validity of the main clinical tests used for the diagnosis of lumbar instability in individuals with LBP and to verify their applicability in everyday clinical practice.

**Methods:**

We searched studies of the accuracy and/or reliability of Prone Instability Test (PIT), Passive Lumbar Extension Test (PLE), Aberrant Movements Pattern (AMP), Posterior Shear Test (PST), Active Straight Leg Raise Test (ASLR) and Prone and Supine Bridge Tests (PB and SB) in Medline, Embase, Cinahl, PubMed, and Scopus databases. Only the studies in which each test was investigated by at least one study concerning both the accuracy and the reliability were considered eligible. The quality of the studies was evaluated by QUADAS and QAREL scales.

**Results:**

Six papers considering 333 LBP patients were included. The PLE was the most accurate and informative clinical test, with high sensitivity (0.84, 95% CI: 0.69 - 0.91) and high specificity (0.90, 95% CI: 0.85 -0.97).

The diagnostic accuracy of AMP depends on each singular test. The PIT and the PST demonstrated by fair to moderate sensitivity and specificity [PIT sensitivity = 0.71 (95% CI: 0.51 - 0.83), PIT specificity = 0.57 (95% CI: 039 - 0.78); PST sensitivity = 0.50 (95% CI: 0.41 - 0.76), PST specificity = 0.48 (95% CI: 0.22 - 0.58)].

The PLE showed a good reliability (k = 0.76), but this result comes from a single study. The inter-rater reliability of the PIT ranged by slight (k = 0.10 and 0.04), to good (k = 0.87).

The inter-rater reliability of the AMP ranged by slight (k = −0.07) to moderate (k = 0.64), whereas the inter-rater reliability of the PST was fair (k = 0.27).

**Conclusions:**

The data from the studies provided information on the methods used and suggest that PLE is the most appropriate tests to detect lumbar instability in specific LBP. However, due to the lack of available papers on other lumbar conditions, these findings should be confirmed with studies on non-specific LBP patients.

**Electronic supplementary material:**

The online version of this article (doi:10.1186/s12998-015-0058-7) contains supplementary material, which is available to authorized users.

## Background

Low back pain (LBP) is a growing health problem in the industrialized world. Despite the high medical expenses required for its management, the prevalence of LBP is increasing [1]. LBP is a heterogeneous condition, and the identification of different sub-groups could help the management decisions [2,3]. One of these sub-groups is lumbar segmental instability [4,5].

The radiologically determined instability is characterized by a loss of passive integrity, causing excessive vertebral translation or rotation. The maximum lumbar flexion-extension radiographs in standing position are considered to be a reference standard to detect the function of the passive stabilization system [6,7]. This imaging method is commonly used to evaluate lumbar segmental mobility in isthmic and degenerative spondylolisthesis and degenerative disc dysfunctions. The radiographic diagnosis of spondylolisthesis is considered to be one of the most efficient methods of identifying lumbar instability [8].

Some authors refer to the concept of instability also considering the so-called “clinical” or “functional” instability, in which no defect of the body architecture of the lumbar spine, and no excessive detectable translation or rotation are shown. However, a poor trunk muscle function and/or an insufficient motor control is believed to be a factor in abnormal inter-segmental movement and LBP [9-11]. Despite this type of instability has not been demonstrated enough as a clinical entity and is not really measureable by any gold standard, it is one of the most frequent fields of interest for chiropractors and manual therapists.

Clinicians have used several clinical tests to detect the spinal instability and/or the ability of the muscles to stabilize the lumbar spine [12]. Recently, some of these tests have been suggested in the “Clinical Practice Guidelines linked to the International Classification of Functioning, Disability and Health from the Orthopaedic Section of the American Physical Therapy Association”, to assess the impairments of body functions in LBP [5]. The most commonly used tests are the Prone Instability Test (PIT), the Passive Lumbar Extension (PLE) test, the Aberrant Movements Pattern (AMP), the Posterior Shear Test (PST), the Prone Bridge Test (PBT), the Supine Bridge Test (SBT), and the Active Straight Leg Raise Test.

Previous reviews separately investigated the diagnostic accuracy [13] or the reliability [14] of the instability tests, but a complete vision about their diagnostic validity to detect lumbar instability is lacking. A single literature review on both the diagnostic accuracy (sensitivity, specificity and likelihood ratios) and the inter-rater reliability of these clinical tests does not exist. More specifically, a researcher could be interested in investigating the reliability of the tests that previously demonstrated sufficient face validity.

The objective of this literature review was to assess the methods used for diagnosis (primarily the accuracy with additional reporting of reliability of these tests) of the clinical tests for lumbar instability in individuals with LBP and investigate their applicability in daily practice.

## Methods

This is a literature review of all the studies presenting a diagnosis of the clinical tests for lumbar instability in individuals with LBP in literature. PRISMA Guidelines [15] were followed during the design, search and reporting stages of this review on diagnostic test studies.

### Literature search

A literature search of relevant literature was performed from July 2012 to December 2013. A comprehensive search, limited to articles in English, Italian and Spanish, was conducted in the following databases: Medline, Embase, Cinahl, PubMed, Scopus. Diagnostic test studies regarding humans published between 1972 and December 2013 were included. Narrative or systematic reviews, guidelines and meta-analyses were excluded.

**Figure 1:**
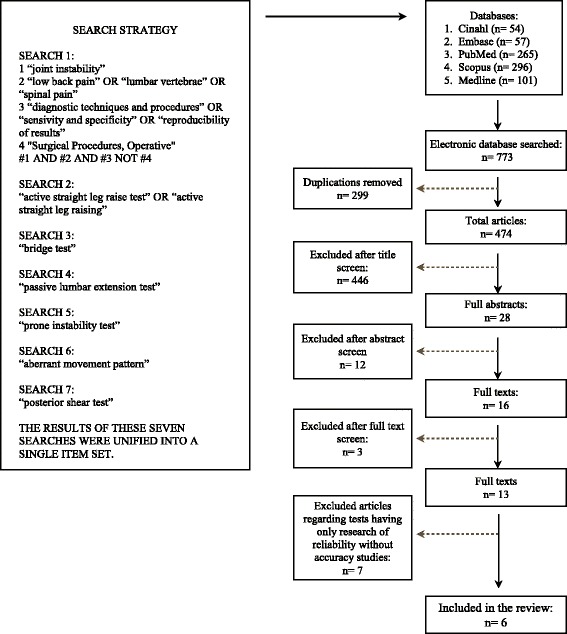
**Flow chart.**

Two authors (SF and TM) independently performed two different and parallel searches to avoid leaving out relevant articles. The search strategies are shown in Figure 1.

The results of these seven searches were unified into a single item set. From the results of the initial search, double citations were removed and then the titles, abstracts and full texts of retrieved articles were independently evaluated for definitive inclusion. When the two reviewers were unable to reach a consensus, a third reviewer (CV) was consulted. In addition to the Internet-assisted search, references were pulled from a textbook on diagnostic accuracy of orthopedic clinical tests [16], and from reference lists of included studies. Finally, an independent hand search including scanning of reference lists from other systematic reviews [13,14] was performed.

### Study selection

Several criteria were used to select eligible studies. Articles examining clinical tests for lumbar instability were included if they met the following criteria:

1. Diagnostic accuracy studies on adult population with sub-acute or chronic LBP were considered if clinical instability tests were employed as index tests. Dynamic radiographs were the reference test to diagnose lumbar instability. The subject articles had to report data which would allow computation of parametric statistical tests of diagnostic accuracy [sensitivity, specificity, or positive and negative likelihood ratios (+LR and -LR)].
2. Reliability studies on healthy or LBP adult population were considered if they concerned the use of clinical tests to diagnose lumbar instability by one or more clinicians. Articles had to report the parametric statistical tests of relationship or agreement.
3. Finally, only the studies in which each test was investigated by at least one study concerning both the accuracy and the reliability were considered eligible.

### Data extraction and quality assessment

One author (TM) gathered data regarding clinical tests, with its description and score, study population (e.g. age, gender, setting, clinical characteristics), inclusion and exclusion criteria, diagnostic reference standard, differences in operationalizing the index tests, study raters. Study results about sensitivity, specificity, LR+, LR-, and reliability were collected (or calculated, if included articles did not provide these data). Other authors (SF and FB) verified data extraction once completed. The methodological quality of included articles was independently assessed by 2 reviewers (TM and FB), using different tools for the 2 types of studies: the Quality Assessment of Diagnostic Accuracy Studies (QUADAS) tool for diagnostic accuracy articles [17] and the Quality Appraisal of Reliability Studies (QAREL) checklist for diagnostic reliability articles [18].

### Data synthesis and analysis

Kappa statistics were used to assess agreement between the 2 raters on article selection and QUADAS and QAREL ratings [19]. The QUADAS and QAREL statement delineates essential items to be reported in diagnostic test studies (Table 1 and Table 2).

**Table 1:** **QUADAS (Quality Assessment of Diagnostic Accuracy Study) tool results**

**Item**		***Fritz*** **et al. [** 24 **]**	***Kasai*** **et al. [** 25 **]**
1.	Was the spectrum of patients representative of the patients who will receive the test in practice?	Y	U
2.	Were selection criteria clearly described?	Y	N
3.	Is the reference standard likely to correctly classify the target condition?	Y	Y
4.	Is the time period between reference standard and index test short enough to the reasonably sure that the target condition did not change between the two tests?	Y	U
5.	Did the whole sample or a random selection of the sample, receive verification using a reference standard of diagnosis?	Y	Y
6.	Did patients receive the same reference standard regardless of the index result?	Y	Y
7.	Was the reference standard independent of the index (i.e. The index test did not form part of the reference standard)?	Y	Y
8.	Was the execution of the index described in sufficient detail to permit replication of the test?	Y	Y
9.	Was the execution of the reference standard described in sufficient detail to permit its replication?	Y	U
10.	Were the index test results interpreted without knowledge of the result of the reference standard?	Y	Y
11.	Were the reference standard results interpreted without knowledge of the results of the index test?	Y	Y
12.	Were the same clinical data available when test results were interpreted as would be available when the test is used in practice?	Y	Y
13.	Were uninterpretable/intermediate test results reported?	Y	Y
14.	Were withdrawals from the study explained?	Y	Y

*Legend: Y = yes, N = no, U = unclear.*

**Table 2:** **QAREL application results**

**Item**		***Hicks*** **et al. [** 23 **]**	***Fritz*** **et al. [** 24 **]**	***Schneider*** **et al. [** 27 **]**	***Ravenna*** **et al. [** 26 **]**	***Rabin*** **et al. [** 12 **]**
1.	Was the test evaluated in a sample of subjects who were representative of those to whom the authors intended the results to be applied?	Y	Y	Y	Y	Y
2.	Was the test performed by raters who were representative of those to whom the authors intended the results to be applied?	Y	U	Y	Y	Y
3.	Were raters blinded to the findings of the other raters during the study?	Y	Y	Y	Y	Y
4.	Were raters blinded to their own prior findings of the test under evaluation?	*N/A*	*N/A*	*N/A*	*N/A*	*N/A*
5.	Were raters blinded to the results of the accepted reference standard or disease status for the target disorder (or variable) being evaluated?	*N/A*	Y	*N/A*	*N/A*	U
6.	Were raters blinded to clinical information that was not intended to be provided as part of the testing procedures or study design?	U	U	Y	N	N
7.	Were raters blinded to additional cues that were not part of the test?	U	U	U	U	U
8.	Was the order of examination varied?	N	Y	Y	Y	N
9.	Was the stability (or theoretical stability) of the variable being measured taken into account when determining the suitability of the time-interval between repeated measures?	(PIT) N	(AMP) Y	N	N	N
10.	Was the test applied correctly and interpreted appropriately?	Y	Y	Y	N	Y
11.	Were appropriate statistical measures of agreement used?	Y	Y	Y	N	U

*Y = yes, N = no, U = unclear, N/A = not applicable; PIT = Prone Instability Test, AMP = Aberrant Movement Pattern.*

Concerning sensitivity and specificity, the acceptable levels were set between 50% (unacceptable test) and 100% (perfect test) [20]. The diagnostic accuracy was considered satisfactory, thus affecting the probability of lumbar instability, with + LR ≥ 2.0 or - LR ≤0.50 [21].

Concerning reliability, the following criteria has been used to determine the strength of the coefficients: ≤ 0.25 = little or no relationship; 0.26 – 0.50 = fair degree of relationship; 0.51 – 0.75 = moderate to good relationship; 0.76 – 1.00 = good to excellent relationship [22].

## Results

Figure 1 shows the process of study selection. Initial searching identified 773 citations. Following the first screening, 299 articles were excluded and 474 citations were retained for the second screening; after reviewing the titles, 446 were excluded and 28 considered of interest, looking at the abstracts 16 were maintained and 13 retrieved in full text. Using the inclusion and exclusion criteria a further 7 articles were excluded. This study finally included 6 papers, considering 333 LBP patients, for the review [12,23-27].

### Quality scores

Two articles of the 6 studies (33%) were identified as having high methodological rigor according to the QUADAS tool (Table 1). Table 2 shows the distribution of studies according to the scores obtained from the assessment of their methodological quality, following the QAREL tool.

### Diagnostic accuracy of the tests

The diagnostic accuracy was investigated by 2 authors only: Fritz et al. [24] and Kasai et al. [25] Four lumbar instability tests were considered: the PLE test, the PIT, the AMP, and the PST. The main characteristics of the studies on diagnostic accuracy are shown in Table 3, whereas Table 4 shows the results.

**Table 3:** **Summary of the studies on diagnostic accuracy**

**Article**	**Clinical tests, scores**	**Inclusion (I) and exclusion (E) criteria**	**Population**	**Reference standard and positive criteria**	**Rater/s**
*Fritz* et al. [24]	**-** **Aberrant Movement Pattern** (Painful arc on flexion, painful arc on return, instability catch, Gower sign, reverse lumbopelvic rhythm). *Positive test: at least 1 of the 5 signs was present.*	I: LBP with or without referred pain on the lower extremities, < 60 yrs	N.49	Dynamic X-ray: the patient stands at the edge of a tall stool with feet flat on the floor and arms folded across the chest. The patient is instructed to flex forward as far as possible for the flexion X-Ray. For the extension X-ray, the patient stands with arms folded, and is asked to extend as far as possible.	1 Physical Therapist
	**-** **Prone instability test** *Positive test: pain provoked during the first part of the test decreases when the test is repeated with the legs off the floor.*	E: contraindications to radiographic assessment (e.g., current pregnancy), previous lumbar fusion surgery, inability (e.g., pain or muscle spasm) to actively flex and extend the spine adequately to permit an assessment of segmental motion	**-** Age: 39.2 ± 11.3 yrs	Criteria for instability: sagittal plane translation greater than 4.5 mm or greater than 15% of the vertebral body width, or sagittal plane rotation greater than 15° at L1/L2, L2/L3, L3/L4 levels, greater than 20° at L4/L5, or greater than 25° at L5/S1.	
	**-** **Posterior Shear Test** *Positive test: familiar symptoms are provoked.*		**-** Duration of symptoms (median days) 78	*Instability diagnosis: 2 segments with either rotational or translational instability OR 1 segment with both translational and rotational instability*	
			**-** Distribution of symptoms: back/buttock only 63.3%, symptoms distal to the knee 30.6%		
			**-** Previous history of LBP: 83.7%		
			**-** LBP episodes becoming more frequent: 30.6%		
*Kasai* et al. [25]	**-** **Passive lumbar extension test:** The subject was in the prone position; both lower extremities the were elevated concurrently to a height of about 30 cm from the bed while maintaining the knees extended and gently pulling the legs. Positive test when the subject complained of strong pain in the lumbar region (“low back pain”, “very heavy feeling on the low back”, “feeling as if the low back was coming off”) during elevation of both lower legs, and such pain disappeared when they returned to the initial position. In contrast, when the subject complained of an abonrmal sensation (mild numbness or prickling sensation) the test was negative.	I: lumbar degenerative diseases	N. 122 subjects with lumbar degenerative diseases: 89 lumbar spinal canal stenosis; 21 lumbar spondylolisthesis; 12 lumbar degenerative scoliosis.	Dynamic x-ray: flexion-extension films of the lumbar spine, lateral vision.	n°3 Orthopedics
		E: /	**-** 39 ± 8.8 yrs;	3 criteria to asses radiological instability: angular motion > 20°; transactional motion > 5 mm; cutoff value of - 5° for the intervertebral endplate angle on the flexion film.	n°2 for testing PLE test (who had 12 and 15 yrs of clinical experience)
			**-** mean illenss duration 11.2 months;	*Radiograph instability: positive for 1 o more of the 3 criteria.*	n°1 for testing Instability catch sign (with 20 yrs of clinical experience).
	**-** **Instability catch sign:** The subject was asked to bend his body forward as much as possible and then return to the erect position; subject who was not able to return to erect position because of sudden low back pain was judged positive to the test.		**-** Complain of pain: 70.5% lumbago, 60.7% intermittent claudicatio, 42.6% neurological symptoms in the lower legs		*For RX evaluation:*
					*n°2 Orthopedics who had 8 and 14 yrs of clinical experience.*

Legend: / = data no present in the article.

**Table 4:** **Results of diagnostic accuracy studies**

**Test**	**2x2 table**		**Sensitivity**	**Specificity**	**PPV**	**NPV**	**+LR (95% CI)**	**-LR (95% CI)**
	**TP**	**FP**						
	**FN**	**TN**						
PIT [23]	20	9	71.4	57.1	69.0	60.0	1.67	0.50
	8	12					(0.97-2.88)	(0.97-2.88)
PLE [24]	32	8	84.2	90.5	80.0	92.7	8.84	0.18
	6	76					(4.51-17.34)	(0.08-0.37)
AMP [23]	5	1	17.9	95.2	83.3	46.5	3.75	0.86
	23	20					(0.47-29.75)	(0.71-1.05)
ICS [24]	10	12	26.3	85.7	45.5	72.0	1.84	0.86
	28	72					(0.87-3.89)	(0.87-1.06)
PCS [24]	14	23	36.8	72.6	37.8	71.8	1.35	0.87
	24	61					(0.78-2.32)	(0.66-1.15)
AS [24]	7	10	18.4	88.1	41.2	70.5	1.55	0.93
	31	74					(0.64-3.76)	(0.78-1.1)
PST [23]	16	11	50.0	47.6	59.3	36.5	0.96	1.05
	16	10					(0.56-1.63)	(0.60-1.85)

T: True, F: False, P: Positive, N: Negative; PPV = Positive Predictive Value; NPV = Negative Predictive Value; +LR = Positive Likelihood Ratio; −LR = Negative Likelihood Ratio; PIT = Prone Instability Test; PLE = Passive Lumbar Extension Test; AMP = Aberrant Movements Sign; ICS: Instability Catch Sign; PCS: Painful Catch Sign; AS: Apprehension Sign; PST = Posterior Shear Test.

Kasai et al. [25] found that the PLE test was the most accurate clinical test, with high sensitivity (0.84, 95% CI: 0.7 - 0.93) and specificity (0.90, 95% CI: 0.82 - 0.95), in a sample of subjects diagnosed with spinal stenosis or lumbar spondylolisthesis or lumbar degenerative scoliosis. The positive and negative LR’s were informative.

The diagnostic accuracy of AMP depends on each singular test. Low sensitivity (0.26, 95% CI: 0.15 - 0.42) and good specificity (0.86, 95% CI: 0.77 - 0.92) were found by Kasai et al. [25] for the Instability Catch Signs. The Painful Catch Sign and the Apprehension Sign showed the same trend, low sensitivity (0.37, 95% CI: 0.24 - 0.54 and 0.18, 95% CI: 0.22 - 0.64 respectively) and good specificity (0.73, 95% CI: 0.61 - 0.8 and 0.88, 95% CI: 0.61 - 0.78 respectively). These tests are included in the AMP, also studied by Fritz et al. [24], who reported low sensitivity (0.18, 95% CI: 0.08 - 0.36) and high specificity (0.95, 95% CI: 0.77 - 0.99) for the AMP test in a cohort of patients with chronic LBP.

The article by Fritz et al. [24] is the only one that studied the diagnostic accuracy of the PIT and the PST. Both tests demonstrated by fair to moderate diagnostic test accuracy. PIT sensitivity = 0.71 (95% CI: 0.53 - 0.85); specificity = 0.57 (95% CI: 0.37 - 0.76); PST sensitivity = 0.50 (95% CI: 0.34 - 0.66); specificity = 0.48 (95% CI: 0.28 - 0.68).

### Reliability of the tests

The reliability of the four clinical tests was studied in 5 papers [12,23,24,26,27]. The main characteristics of the studies on reliability and their results are shown in Table 5, whereas Table 6 shows the results in terms of inter-rater reliability.

**Table 5:** **Summary of the articles on reliability**

**Article**	**Clinical test and scores**	**Inclusion (I) and exclusion (E) criteria**	**Population**	**Reliability**	**Rater/s**
*Hicks* et al. [23]	**- Painful arc in flexion**	I: current complaints of LBP.	N 63	Inter-rater reliability.	N. 4
	**- Painful arc on return**	E: symptoms referred below the knee, LBP which may be attributed to current pregnancy, fractures in acute phase, tumor, infection, previous lumbar surgical fusion.	20-66 yrs	For each pair of raters, the first rater performs all clinical examination measures on each subject; the second rater, who is blinded to the results of the first evaluation, then performs the same examination procedures, after a minimum of 15- minutes.	PT1: PT and chiropractor with 3 yrs of experience as a chiropractor and 2 yrs as an OMT
	**- Instability catch**		- Age 36.0 ± 10.3		PT2: PT with 6 yrs of experience in orthopedic setting
	**- Gower sign (“thigh climbing”)**		- Gender: 38♀, 25♂		PT3: OMT with 8 yrs of experience
	**- Reversal of lumbopelvic rhythm**		- Previous LBP episodes, 51/63.		PT4: PT with 4 yrs of experience on orthopedic environment.
	**-** **Aberrant Movement Pattern:** positive if at least one of the five previously cited signs is present.				*3 pair of raters: PT1 + PT2, PT2 + PT3, PT1 + PT4*
	**-** **Prone Instability Test:** *Positive test: pain provoked during the first part of the test disappears when the test is repeated with the legs off the floor.*				
*Fritz* et al. [24]	**-** **Aberrant Movement Pattern:** Painful arc on flexion; Painful arc on return; Instability catch; Gower sign (“thigh climbing”); Reverse lumbopelvic rhythm. *Positive test when at least 1 of the previous 5 signs was present.*	I: complaint of LBP with or without radiation into the lower extremities, < 60 yrs	N. 38 patients taken by a sample of 49 patients with these characteristics:	Inter-rater reliability.	N. 2 physical therapists
	**-** **Prone Instability Test:** *Positive test when pain provoked during the first part of the test decreases when the test is repeated with the legs off the floor.*	E: contraindications to radiographic assessment (e.g., current pregnancy), previous lumbar fusion surgery, inability (e.g. pain or muscle spasm) to actively flex and extend the spine adequately to permit an assessment of segmental motion.	- Age: 39.2 ± 11.3 yrs;	The second rater repeats the assessment 5 minutes after the first rater’s assessment	
	**-** **Posterior Shear Test:** *Positive test if familiar symptoms are provoked.*		- Duration of symptoms (median days) 78;		
			- Distribution of symptoms: back/buttock only 63.3%, symptoms distal to the knee 30.6%;		
			- Previous history of LBP: 83.7%		
			- LBP episodes becoming more frequent: 30.6%		
*Schneider* et al. [27]	**-** **Prone instability test:** *Positive test when pain provoked during the first part of the test disappears when the test is repeated with the legs off the floor.*	I: History of LBP, age between 18 and 65 years, ability to tolerate lying prone	N. 39 volunteer patients with history of LBP and undergoing chiropractic treatment at the time of their enrollment in the study	Inter-rater reliability.	N. 2 experienced doctors of chiropractic (25 and 10 years of clinical experience, respectively).
		E: History of prior lumbar surgery, stenosis, scoliosis greater than 20°, unstable spondylolisthesis, positive nerve root tension or radiculopathy, any red flags suggestive of spinal pathology.			
*Ravenna* et al. [26]	**-** **Prone Instability Test with additional guidelines:**	I: chronic or recurrent LBP; age 18 to 60 years; current symptoms of LBP, but not acute phase.	- N. 30	● Inter-rater reliability for PIT examined under 2 conditions:	N. 2 examiners:
	→ A trunk stabilizing belt is placed around the subject and the table at shoulder level,	E: BMI > 30 kg/m^2^, disk herniation, symptoms referred below the knee, lower extremity weakness or loss of reflexes, history of spinal surgery or fracture, spinal deformity, systemic inflammatory condition, neurologic disease or other serious medical conditions. LBP attributable to pregnancy or a primary hip problem.	- Age 36.1 ± 11.8 yrs	*●* PIT test with additional guidelines	Second-year physical therapy student
	→ A stool may be placed under the subject’s feet if the feet do not comfortably reach the floor.		- Men: 56.7%	*●* PIT test without additional guidelines.	Licensed physical therapist with 2 years of clinical experience in outpatient orthopedic physical therapy
			- Diagnosis: degenerative disk disease 16.6%, disk problem 10%, LBP 73.4%		
			- Previous LBP episodes: 83.0%		
			- Current VAS (0–10): 2.8 ± 1.6		
	*Positive and negative criteria:*				
	*● Positive level if the subject reports a decrease of pain with the second P/A, lifting the legs in the second part of the test*				
	*● Negative test if the subject reports superficial bone-on-bone pressure;*				
	*● Negative test if the subject reports an increase in symptoms lifting the legs during the second part of the test;*				
	*● Negative level if the subject reports an increase or same with the second P/A, compared with the first.*				
*Rabin* et al. [12]	**-** **Aberrant Movement Pattern.** Painful arc on flexion; Painful arc on return; Instability catch; Gower sign (“thigh climbing”); Reverse lumbopelvic rhythm. *Positive test when at least one of the cited five signs is present.*	I: age between 18 and 60 years, main complaint of LBP and/or related leg symptoms (i.e., pain, paresthesia)	N. 30 consecutive patients with LBP of any duration, with or without associated leg symptoms.	Interrater reliability	N. 4 raters physical therapists, with experience ranging from 13 to 25 yrs.
	**-** **Prone Instability Test:** *Positive when pain elicited during the first part of the test is relieved or abolished during the second part.*	E: pregnancy; history suggesting a non-mechanical origin of symptoms (e.g., malignancy, inflammatory conditions), LBP due to a fracture, osteoporosis, regular use of corticosteroids, rheumatoid arthritis, presence of 2 or more signs suggesting lumbar nerve root compression.	**-** Age: 33.5 ± 8.0 yrs	AMP was assessed by the two raters simultaneously; PIT and PLE are assessed by the two raters separately (second assessment 5 minutes after the first one).	One rater with postprofessional master’s degree (contributes to rating all subjects).
	**-** **Passive Lumbar Extension Test:** *Positive if LBP is elicited.*		**-** Gender: 15♀, 15♂		Other raters with bachelor degree in physical therapy contribute to rating in 23, 4, and 3 subjects, respectively.
			**-** Duration of symptoms: 164.4 ± 321.8 days		
			**-** Previous LBP episodes: 20 subjects

**Table 6:** **Summary of results on reliability**

**Article**	**Test**	**Reliability**	**Results**
Hicks et al. [23]	Aberrant Movement Pattern	Inter-rater reliability	k = 0.60 (95% CI: 0.44; 0.73)
	Prone Instability Test		k = 0.87 (95% CI: 0.80; 0.94)
Fritz et al. [24]	Aberrant Movement Pattern	Inter-rater reliability	k = −0.07 (95% CI: −0.45; 0.31)
	Prone Instability Test		k = 0.69 (95% CI: 0.59; 0.79)
	Posterior Shear Test		k = 0.27 (95% CI: 0.14; 0.41)
Schneider et al. [27]	Prone Instability Test	Inter-rater reliability	k = 0.46 (95% CI: 0.15, 0.77)
			k weighed = 0.58
Ravenna et al. [26]	Prone Instability Test with additional guidelines	Inter-rater reliability	(With*) k = 0.10 (95% IC: −0.27; 0.47)
			k weighed = 0.27 (95% IC: −0.08; 0.61)
			(Without*) k = 0.04 (95% IC: −0.34; 0.42)
			k weighed = 0.47 (95% IC: 0.15; 0.78)
Rabin et al. [12]	Aberrant Movement Pattern	Inter-rater reliability	k = 0.64 (95% IC 0.32; 0.90)
	Prone Instability Test		k = 0.67 (95% IC 0.29; 1.00)
	Passive Lumbar Extension test		k = 0.76 (95% IC 0.46; 1.00)
	Active Straight Leg Raising		k = 0.53 (95% IC 0.2; 0.84)

* = Additional guidelines.

The PLE test showed a better reliability, but this result comes from a single study [12]. The inter-rater reliability of this test resulted good (k = 0.76).

Five studies investigated the inter-rater reliability of the PIT. This reliability was considered fair by Schneider et al. [27] (k = 0.46) and Ravenna et al. [26] (k = 0.10 and 0.04), moderate by Fritz et al. [24] and Rabin et al. [12] (k = 0.69 and k = 0.67, respectively), and good by Hicks et al. [23] (k = 0.87).

The inter-rater reliability of the AMP was studied by Hicks et al. [23] Fritz et al. [24] and Rabin et al. [12]. Whereas Fritz et al. [24] found poor reproducibility (k = −0.07), Hicks et al. [23] (k = 0.60) and Rabin et al. [12] (k = 0.64) calculated moderate reliability. The inter-rater reliability of the Posterior Shear Test was only studied by Fritz et al. [24] showing poor reliability (k = 0.27).

### Implications for clinical practice

The data from the studies provided information on the tests and methods used, the error of measurement and also the validity of the tests. However, only 5 studies (83.3%) provided information concerning the setting and the years of raters clinical experience, whereas all studies identified the person performing the assessment and his/her professional competence.

## Discussion

This literature review was aimed to identify the most reliable findings concerning the assessment of methods for diagnosis of the clinical tests for lumbar instability in LBP subjects.

The lumbar instability is traditionally a field of debate. Lumbar segmental instability in the absence of defects of the bony architecture of the lumbar spine has also been cited as a significant cause of chronic low back pain [5,28]. The differences between surgical instability criteria and “functional instability” criteria were defined by Panjabi [29] decades ago. Chiropractics and Manual Therapists are more interested in the lost of motor control than in hypermobility detectable with flexion/extension radiological imaging, which is more useful to spine surgeons. However, the difficulty to clinically detect abnormal or excessive inter-segmental motion makes these tests often insensitive and unreliable and it becomes a limit for the clinical diagnosis of lumbar segmental instability [30,31]. The lack of studies in this field emerges also by our research, which found many studies about reliability of tests used by clinicians but few about their accuracy. Being aware that this criterion is too rigorous for manual therapists we have chosen to be rigorous and we have been forced to do our research having as reference the best reference (gold standard) to instability, that is dynamic X-rays. The result is that many other tests used in the manual clinical practice to detect lumbar clinical instability (i.e. active hip abduction test or hip extension test) have not been considered because no study had investigated their accuracy. These tests are not present in this review, so that, in latest analysis, our study could be considered as a literature review of accuracy of lumbar clinical tests with additional reporting of reliability information.

Six high-quality studies were selected and four lumbar clinical instability tests (PLE test, PIT, AMP and PST) satisfied the inclusion criteria.

### Accuracy

The characteristics of the samples of the 2 subject studies [24,25] cannot be considered accurate. Fritz et al. [24] studied a population whose majority had a prior history of LBP, and in which only 30.6% (n = 15) of people complained about distal knee symptoms. Kasai et al. [25], however, investigated a population with specific lumbar conditions (lumbar spinal canal stenosis, lumbar spondylolisthesis or lumbar scoliosis), most of whom had intermittent claudication, and 42.6% (n = 52) had neurological leg symptoms.

The PLE test was the most accurate and informative test, even though it was measured by only one study, in patients affected by lumbar degenerative diseases. Despite the PLE test appears to be a potentially effective clinical test to detect lumbar instability, the characteristics of the investigated sample and the presence of only one study on its diagnostic accuracy may suggest the necessity of studies on non-specific LBP patients.

The PIT demonstrated low to moderate sensitivity and specificity [24] indicating that this test has limited accuracy in diagnosing lumbar instability in patients with LBP.

The PST showed relatively poor sensitivity and specificity [24], indicating that this test is less accurate than the PLE test and the PIT to detect lumbar instability.

The Instability Catch Sign, the Painful Catch Sign and the Apprehension Sign are three of the five signs included in the AMP investigated by Fritz et al. [24]. The relatively low sensitivity and high specificity resulting from the study of Kasai et al. [25] suggest caution in the use of these tests to diagnose lumbar instability. According to Hicks et al. [23], these 5 tests should be used together, as a complete observation of the trunk movement and the 5 signs could be considered as only one comprehensive test. However, positive results on AMP and PIT, which demonstrated moderate sensitivity and specificity, were considered predictive for a favorable response to stabilization exercises [32].

### Reliability

The characteristics of the samples were not always well explained or were not reliable. The PLE test [12] and the PIT [12,23,24] demonstrated good inter-rater reliability. The reliability of PLE test is evident in younger subjects referred to outpatient physical therapy [12]. Five studies on PIT demonstrated very different inter-rater reliability scores. Nevertheless, the 2 studies showing fair reliability [26,27] are affected by possible bias; in the first case [27] due to a very limited sample size and in the second case [26] due to procedures and methodological weaknesses as the involvement of novel raters and the use of a modified test. The main statistical problem was the presence of few samples that could invalidate the k score. Despite all the other 4 studies adopting the PIT closely followed its original description, some differences in the positivity criteria were found. Hicks et al. [23] and Schneider et al. [27] judged the test positive when the pain disappeared in the second part of the test; Fritz et al. [24] when the pain decreased, whilst for Rabin et al. [12] the pain had to be both relieved or abolished.

After having excluded the two studies with the main methodological weaknesses, the reliability of the PIT appeared from moderate to good.

The AMP reliability was investigated in three studies [12,23,24] but their results were not similar and ranged from insufficient reliability [24] to moderate reliability [12,23]. The PST was investigated by only one study and scored the lowest reliability [24], which is insufficient to recommend its use.

### Implications for clinical practice

After an initial inspection of the articles it appears that the information derived from the studies could provide a useful picture of the items that contribute to the definition of “applicability in rehabilitation practice”. Sufficient information was provided on the execution of the tests, whereas little information regarded the duration, and the time needed to process data. Considering that in clinical practice a standard manual therapy session normally lasts 30 minutes, it may be the case that a series of tests proposed in the literature cannot be repeated by the clinicians due to lack of time. The attempt to identify methods for the evaluation of lumbar instability in patients with LBP allowed us to select some tests that are suitable for clinicians in everyday clinical practice. The time needed to test and process data are compatible with clinical practice and research purposes. Starting from the same key-words used for the search of the articles of the literature review, 4 clinical tests (PIT, PLE, AMP and PST) investigated by 2 studies [24,25] met the criteria of applicability in clinical practice.

### Limits

The main limitation of this review is the small number of articles found on any single test. Only 2 studies concerned the diagnostic accuracy, while for the studies investigating the reliability, the results are limited by statistical or methodological weaknesses. For example, the Ravenna’s [26] conclusions should be cautiously interpreted also for some significant modifications made to standardize the PIT, such as the different hip and knee positions, the use of a stabilization scapular belt and a stool for foot placement.

The average age and the characteristics of the spinal dysfunctions of the samples were not homogeneous in the different studies, thus reducing the external validity of the results. Another limitation of this review concerns the insufficient homogeneity regarding the execution and interpretation of the tests. As already mentioned, a lack of standardization of a test affects comparative analyses among different studies and the implementation of that test in clinical practice.

## Conclusions

The actual state of the art of clinical tests for lumbar instability include 6 studies of almost 333 patients and 4 clinical tests. Our data suggest that the PLE test is the most suitable test for detecting lumbar instability, thanks to its excellent diagnostic accuracy, and good reliability. Further studies on the diagnostic properties of the PLE test to detect lumbar instability among different populations with LBP are suggested.

After more than 20 years from the definition of the importance of diagnostic clinical tests for lumbar instability in individuals with LBP, clinicians can use some tests showing encouraging results in terms of accuracy and reliability. Nevertheless, their application in daily practice might be affected by insufficient research and evidence on their performances. Future research should be oriented to compare in the same study different assessment methods on the same sample size, in order to evaluate their reliability and validity.
